# Working memory capacity and (in)voluntary mind wandering

**DOI:** 10.3758/s13423-020-01737-4

**Published:** 2020-04-24

**Authors:** Alexander Soemer, Ulrich Schiefele

**Affiliations:** Department of Psychology, University of Potsdam, Karl-Liebknecht-Str. 24-25, 14476 Potsdam, Germany

**Keywords:** mind wandering, intention, working memory, executive control

## Abstract

According to influential accounts of mind wandering (MW), working memory capacity (WMC) plays a key role in controlling the amount of off-task thought during the execution of a demanding task. Whereas WMC has primarily been associated with reduced levels of *involuntarily* occurring MW episodes in prior research, here we demonstrate for the first time that high-WMC individuals exhibit lower levels of *voluntary* MW. One hundred and eighty participants carried out a demanding reading task and reported their attentional state in response to random thought probes. In addition, participants’ WMC was measured with two common complex span tasks (operation span and symmetry span). As a result, WMC was negatively related to both voluntary and involuntary MW, and the two forms of MW partially mediated the positive effect of WMC on reading performance. Furthermore, the negative relation between voluntary WM and reading remained significant after controlling for interest. Thus, in contrast to prior research suggesting that voluntary MW might be more closely related to motivation rather than WMC, the present results demonstrate that high-WMC individuals tend to limit both involuntary and voluntary MW more strictly than low-WMC individuals.

**Electronic supplementary material:**

The online version of this article (10.3758/s13423-020-01737-4) contains supplementary material, which is available to authorized users.

Most of us have experienced situations in which our thoughts drifted off while carrying out potentially important tasks, such as proofreading a manuscript or making a tax declaration. Although content, emotional valence, as well as temporal focus of such task-unrelated thoughts can be quite diverse, two common characteristics are that these thoughts are not directly related to any given primary activity and that they originate from one’s internal stream of thoughts rather than being cued by external events (Smallwood & Schooler, 2015).^1^

Task-unrelated thoughts have received a great deal of interest in recent psychological research, where they are also being referred to as *mind wandering* (MW). Part of this recent interest follows from the ubiquity of MW in daily life (e.g., Killingsworth & Gilbert, 2010; Unsworth, Brewer, & Spillers, 2012) and its potential impact on our actions. In particular, MW seems to have a negative impact on task performance (e.g., McVay & Kane, 2012; Soemer & Schiefele, 2019), and it is frequently accompanied by negative emotions (Killingsworth & Gilbert, 2010) and cognitive failures (Unsworth et al., 2012).

An important question for MW researchers is why certain individuals more than others frequently engage in MW while carrying out a given primary task. Related to this question, prior research has highlighted the role of working memory as a key predictor of MW (Kane & McVay, 2012). Working memory is thought to underlie a wide variety of cognitive skills, and individual differences in working memory capacity (WMC) are a major predictor for performance in a broad range of tasks (Kane, Bleckley, Conway, & Engle, 2001). According to an influential working memory theory, individual differences in WMC reflect variation in a domain-general executive control capability that is critical for maintaining one’s focus of attention on a primary task and inhibit distraction from both external and internal sources (Kane et al., 2001). Based on this account of working memory, some researchers have argued that the occurrence of MW reflects failures of executive control, whereby personal concerns of an individual overtake the focus of attention and disrupt execution of the primary task (Kane & McVay, 2012; McVay & Kane, 2012). In support of this view, it has been observed that high-WMC individuals report fewer MW episodes than low-WMC individuals while carrying out a demanding task, and this difference partially explains their better task performance (e.g., McVay & Kane, 2012; Robison & Unsworth, 2018).

The control failures account has successfully been used to explain the relations between WMC, MW, and task performance in demanding tasks (e.g., reading comprehension) based on the assumption that individuals normally seek to avoid and suppress MW episodes to successfully perform the tasks; that is, the control failures account considers MW to be something that happens despite one’s best effort to focus on the primary task (Kane & McVay, 2012, pp. 348–349).

However, both personal experience and research suggest that there are situations in which an individual freely chooses to engage in MW instead of focusing on the primary task. This may happen, for example, because one does not expect MW to have a negative impact on task performance, because a task is too demanding or because of a lack of motivation to perform the task (Seli, Cheyne, Xu, Purdon, & Smilek, 2015; Seli, Risko, & Smilek, 2016). Taking into consideration such cases of MW, some researchers have argued for making a distinction between “voluntary” and “involuntary” MW (e.g., Seli et al., 2016).^2^ Here, voluntary MW is defined as some kind of MW that an individual freely chooses to engage in, whereas involuntary MW occurs despite an individual’s best effort to focus on the primary task.

Highlighting the importance of making this distinction, Seli et al. (2015) reported a significant negative correlation between the motivation to perform well on a task and a measure of voluntary (but not involuntary) MW suggesting that one cause for voluntary MW could be low motivation. Robison and Unsworth (2018) replicated this negative association and, furthermore, found an inverse association between involuntary (but not voluntary) MW and a measure of alertness. Finally, Seli et al. (2016) found voluntary and involuntary MW to be differently affected by task difficulty manipulations: Whereas voluntary MW was more frequent when participants carried out an easy version of the Sustained Attention to Response (SART) task compared with a more difficult version, the opposite was true for involuntary MW.

Although prior research suggests that it may be useful to distinguish between voluntary and involuntary MW, it is still not clear how WMC may be related to these two forms of MW. On the one hand, studies on the relation between WMC and MW have so far rarely distinguished between voluntary and involuntary MW and instead implicitly or explicitly characterized MW as involuntary (e.g., Kane & McVay, 2012, p. 348). On the other hand, in those cases where the distinction was made, the resulting evidence is inconclusive. For example, Soemer and Schiefele (2019) found a significant negative association between WMC and involuntary MW, but a nonsignificant association between WMC and voluntary MW with a reading task. The same pattern was found in a study by Robison and Unsworth (2018) with a number of attention-demanding laboratory tasks. Finally, Ju and Lien (2018) reported a significant negative relation between involuntary MW and WMC in a demanding version of a modified *N*-back task but a nonsignificant relation in a nondemanding version of this task. Importantly, there was no statistically significant relation between voluntary MW and WMC in both task conditions.

Although one might be tempted to regard such nonsignificant results as support for the absence of a (measurable) relation between voluntary MW and WMC, it must be noted that statistically nonsignificant results, even if repeatedly demonstrated, generally do not constitute evidence in favor of a null hypothesis (e.g., Nickerson, 2000). For this reason, none of the aforementioned experiments should be taken as evidence for the hypothesis that voluntary MW and WMC are completely unrelated. And also, on theoretical grounds, there are reasons to believe that voluntary MW and WMC might be more strongly related than current evidence suggests. First, low-WMC individuals are known to experience more difficulties while carrying out demanding tasks (e.g., Swanson, Zheng, & Jerman, 2009), and they should therefore be more inclined to skip (difficult) parts of the task, rest, or even give up completely at some point. As a consequence, low-WMC individuals should exhibit a stronger tendency to deliberately start thinking about something unrelated to the task or continue a MW episode that originally started as an involuntary MW episode (i.e., due to an executive control failure).

Second, low-WMC individuals may generally be less motivated to engage or persist in demanding tasks because they do not expect themselves to be good at it (e.g., Linnenbrink & Pintrich, 2003). As a consequence of such low self-efficacy beliefs, they may not invest much of their attentional capacity in a demanding task right from the beginning and instead allow for more voluntary MW.

Third, prior research suggests that low-WMC individuals are less accurate with regard to meta-cognitive judgments of their performance in demanding tasks (e.g., Komori, 2016). Based on such results, one may speculate that low-WMC individuals more easily misjudge the impact of MW on task performance, and may therefore tolerate more voluntary MW compared with high-WMC individuals. Put differently, high-WMC individuals may more strictly limit voluntary MW because they are more aware of the negative consequences on task performance.

These three considerations lead to predicting a *negative* relation between WMC and voluntary MW, given a suitable experimental paradigm and appropriate instruments for measuring both constructs. However, a number of factors might contribute to the failure to demonstrate such a relation. First, some of the aforementioned studies used single-task measures of WMC (Ju & Lien, 2018; Soemer & Schiefele, 2019), raising the possibility that variance related to individual differences in WMC was masked by a large amount of error variance, which, in turn, led to an underestimation of the correlations between WMC and voluntary MW. Second, some of the studies report very low rates of voluntary MW (e.g., between 2% and 4% in Robison & Unsworth, 2018), suggesting floor effects that, again, make it difficult to detect significant correlations between voluntary MW and WMC. Third, the studies have used various tasks with different cognitive demands, a variable that likely moderates the relation between WMC and voluntary MW.^3^ In one study, for example, voluntary MW was measured during the execution of three different laboratory tasks that later showed correlations between voluntary MW and measures of WMC ranging from −.17 to .11 (Robison & Unsworth, 2018). In another case, task difficulty was manipulated within a single task (reading), and this manipulation led to low rates of voluntary MW in the easier conditions (Soemer & Schiefele, 2019) meaning, again, that it was difficult to demonstrate a significant correlation between voluntary MW and WMC.

## The current study

This study set out to investigate the associations between WMC and voluntary and involuntary MW, avoiding the aforementioned pitfalls of prior research and hypothesizing that WMC would be negatively related to both forms of MW for the above outlined reasons. To gather support for this hypothesis, we first measured participants’ WMC with two common complex span tasks—operation span and symmetry span (Conway et al., 2005) and then estimated participants’ individual MW rates during the execution of a demanding primary task. For the present purpose, reading comprehension was our task of choice because of its high ecological validity, its moderate to high difficulty, its frequent use in MW research (e.g., McVay & Kane, 2012; Soemer & Schiefele, 2019; Unsworth & McMillan, 2013), and because comparisons could be made with prior research (Soemer & Schiefele, 2019). Probe-caught experience sampling was used to estimate individual MW rates: Participants were randomly interrupted during reading and asked to indicate whether they had been focusing on the text or engaging in voluntary or involuntary MW prior to the interruption. After reading each text, participants answered comprehension questions. Because MW rates during reading and comprehension usually depend on prior experience with and interest in the topic of a text (e.g., Soemer & Schiefele, 2019), we included measures of topic interest and topic familiarity to be used as control variables in later modeling.

### Hypotheses

Based on prior research (e.g., McVay & Kane, 2012; Soemer & Schiefele, 2019; Unsworth & McMillan, 2013), we predicted that WMC would support comprehension, that WMC would be negatively related to involuntary MW, and that involuntary MW would partially mediate the relation between WMC and comprehension (e.g., Soemer & Schiefele, 2019). The most important prediction, however, concerned the relation between WMC and voluntary MW. We expected this relation to be negative, (1) because we assumed that low-WMC individuals experience more difficulties during reading and therefore more easily skip parts of the text, rest, or give up completely; (2) because they may generally invest less in a task that they believe they are not good at (low self-efficacy beliefs); or (3) because they more easily misjudge the impact of MW on comprehension. Furthermore, we predicted voluntary MW to negatively affect comprehension (cf. Soemer & Schiefele, 2019) and a partial mediation of the effect of WMC on comprehension through voluntary MW.

**Fig. 1:**
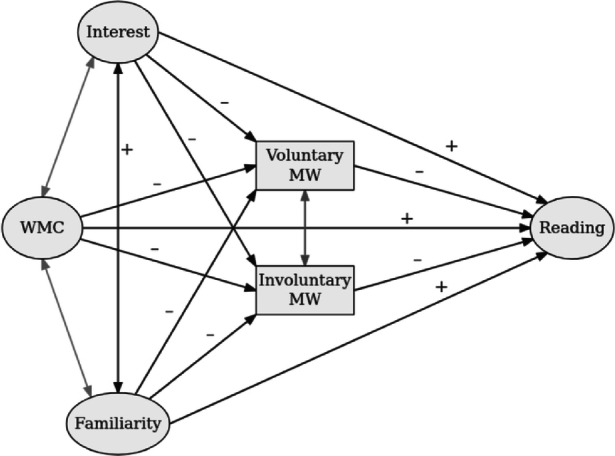
Structural equation model corresponding to our hypotheses. “+” and “–” indicate the to-be-expected signs of the coefficients

Because reading was selected as the primary task in this study, we included the control variables interest and topic familiarity, and we predicted these variables to be positively related to comprehension (Robison & Unsworth, 2018; Soemer & Schiefele, 2019; Unsworth & McMillan, 2013). Furthermore, we predicted negative effects of interest on both forms of MW, in line with prior research (Soemer & Schiefele, 2019). As regards the association between interest and topic familiarity, we predicted a strong positive relation because one usually reads more often about topics that are perceived as interesting (e.g., Alexander, Kulikowich, & Jetton, 1994; Soemer & Schiefele, 2019) and because more familiar topics promote the perception of interest (Schraw, Flowerday, & Lehman, 2001). Finally, we predicted a negative relation between topic familiarity and both forms of MW, although prior research suggests that this relation can be small when interest is controlled for (Soemer & Schiefele, 2019). A visual summary of the predictions can be found in Fig. 1.

## Method

In the following, we will report how we determined our sample size, all data exclusions (if any), all manipulations, and all measures in the study (Simmons, Nelson, & Simonsohn, 2012). The experiment was not preregistered. However, the data and analysis scripts are openly available (https://osf.io/3hdwq/).

All participants were tested individually in a soundproof booth at our lab. At the beginning of the experiment, they received brief instructions by a trained experimenter regarding the tasks that they carried out during the following 90-minutes session. Detailed instructions were given prior to each individual task on the computer screen. The instructions for the reading task not only emphasized that participants were going to take a comprehension test after having read each text but also contained a short paragraph explaining to them that they would be interrupted from time to time during reading and asked about their thoughts prior to the interruption (see the Appendix). To ensure that the participants had understood the instructions, they were allowed to ask questions at the beginning of the session and between each task.

### Participants

One hundred and eighty participants were recruited from the university’s student population (*M*_age_ = 23.35 years, *SD* = 3.93 years, 155 females, 25 males). This sample size was set a priori based on our experience with earlier studies and the fact that several measures were taken to improve estimation of the to-be-measured variables compared with our previous study (Soemer & Schiefele, 2019). There were no additional stopping rules for data collection, which continued over the course of two academic terms until the targeted sample size was reached.^4^ None of the participants had previously participated in any reading or MW experiments at our department, and we therefore assume that all participants were naïve with regard to the purpose of this experiment. After completion of the experiment, they were rewarded with either 12€ or course credits.

### Instruments

#### Working memory capacity

Participants carried out two commonly used complex span tasks (Conway et al., 2005): operation span and symmetry span. Both tasks required participants to maintain a set of items while making true/false judgments on a separate set of items. The two parts of the tasks were interleaved such that participants first saw a to-be-stored item (900 ms on, 100 ms off), then saw a second item on which they made a true/false judgment via button press (2,000 ms time-out), then saw the next to-be-stored item, and so on. The alternation of storage and processing episodes ended after three to seven cycles. Participants were then requested to recall the stored items in their correct serial order. The recall score was derived by dividing the number of items recalled in the correct position of the presented sequence by sequence length (partial credit scoring; see Conway et al., 2005).

The major difference between the two span tasks concerned the nature of the items. In the operation-span task, the to-be-stored items consisted of single monosyllabic letters, whereas the processing episodes consisted of math equations taking the form *(a ± b) × c* = *d*. Participants had to remember the letters and verify the correctness of the equations in between letter presentation. At recall, both letters that were part of the sequence and letters that were not, but belonged to the item pool, appeared on the screen. Participants had to recall the letter sequence by clicking on the letters one after another. In the symmetry-span task, the to-be-stored items consisted of square locations in a 4 × 4 grid, whereas the processing task required them to judge the vertical symmetry of 32 × 32 matrix patterns. At recall, an empty grid appeared, and participants had to recall the sequence of locations by clicking on the corresponding cells of the grid.

Prior to each task, participants were instructed to perform the processing part as quickly and as accurately as possible. Furthermore, we measured baseline processing speed for the equation and symmetry verification tasks within a single-task condition prior to administering the main tasks. This was done to check whether individuals strategically slow down their processing speed in the later dual-task trials. Processing times deviating more than two standard deviations from the mean times in the single-task trials were taken as an implicit filter criterion for the dual-task trials. Whenever a participant exceeded this individual criterion in a trial, a message appeared on the screen after recall, reminding the participant to perform the processing task as quickly and as accurately as possible. In addition, trials with processing accuracies below 80% were not considered in the later analyses, because this suggests that participants did not sufficiently focus on the processing part of the task (Conway et al., 2005).

Prior to the main session, participants were given the opportunity to practice the memory and processing parts both separately and together. In the main session, all participants completed four trials of different sequence lengths, starting with the three items and progressing up to the seven items. Final span scores were calculated by averaging across trials.

#### Reading comprehension

After completing the complex span tasks, participants read three texts on a computer screen and answered comprehension questions after each text. The texts were taken from the study of Soemer and Schiefele (2019) and dealt with (1) the physics behind global airflows, (2) stock market bubbles, and (3) semantic knowledge representation. They were edited to be of moderate readability as measured with the readability index ‘LIX’ (Björnsson, 1968). The texts were between 913 and 1,281 words long. Each text was displayed page wise, around 150 to 250 words per page, and typed in a dark gray serif font on a white background. To make reading as natural as possible, participants were allowed to freely navigate through the pages by pressing the left-arrow and right-arrow keys on a keyboard. There was no time limit for reading a single text. However, all three texts had to be completed within the overall session limit of 90 minutes.

Each comprehension test consisted of nine 4-item multiple-choice questions per text and was administered after reading (and after rating interest and topic familiarity). Participants were allowed to take as much time as necessary to provide the answers. The test questions were constructed as to measure fact knowledge and required the participants to complete a sentence with one out of four highly similar response options. For example, one test question for the “stock market bubbles” text was “*A central bank can exacerbate speculation on credit by . . . ,*” and the four response options were *“lending money to private banks at a low interest rate”* (correct option), “*lending money to private banks at a high interest rate,”* “*lending money to private banks over a short period,”* and “*lending money to private banks over a long period.”* Each correct answer was assigned 1 point.

#### Mind wandering

Participants were randomly interrupted during reading after a random interval between 60 to 90 seconds and were required to respond with one of three options: *“I was thinking about something related to the text*,” *“I was voluntarily thinking about something unrelated to the text*,” and *“I was involuntarily thinking about something unrelated to the text*.*”* Participants were instructed that they should make their response based on whether the thoughts they were engaging in prior to the interruption were aimed at promoting comprehension. The distinction between voluntary and involuntary MW was made clear to them prior to the experiment (see the Appendix). Because the number of probes that an individual received during reading could vary across participants and texts, individual MW rates per individual and texts were calculated by dividing the responses to the probes by the number of probes.

#### Interest

Participants rated their level of interest on the items *“I found this text interesting,” “I like to read about such topics,”* and *“I found the topics in this text very stimulating.”* Ratings were to be provided on a 4-point scale ranging from 0 (*strongly disagree*) to 3 (*strongly agree*).

#### Topic familiarity

Participants rated their levels of topic familiarity on the items *“I have read or heard about these things many times before,” “My prior knowledge helped a lot to understand this text,”* and *“I am familiar with the topics of this text.”* Ratings were to be provided on a 4-point scale ranging from 0 (*strongly disagree*) to 3 (*strongly agree*).

## Results

The data of one participant had to be discarded because this participant’s reading times were shorter than 1 minute per text, suggesting that he or she had not properly read the texts. Descriptive and inferential statistics were computed using the data of the remaining 179 participants.

### Descriptive statistics

Correlations between the measures were mostly weak to moderate and followed the expected direction (see Table 1 for latent correlations and Table 4 for manifest correlations). In particular, WMC, interest and topic familiarity were positively correlated with comprehension, whereas both forms of MW were negatively correlated with comprehension, interest, and topic familiarity. Most importantly, the correlations between WMC and voluntary/involuntary MW were both negative, as predicted.

**Table 1:** Latent bivariate correlations and descriptive statistics for the investigated constructs

	Interest	Topic familiarity	WMC	Voluntary MW	Involuntary MW	Comprehension
Interest						
Topic familiarity	0.52^***^					
WMC	0.08	0.08				
Voluntary MW	−0.32^***^	−0.15^***^	−0.13^*^			
Involuntary MW	−0.29^***^	−0.18^***^	−0.16^*^	−0.15^*^		
Comprehension	0.47^***^	0.27^***^	0.35^***^	−0.39^***^	−0.32^***^	
Mean	1.69	1.31	0.70	0.13	0.27	1.81
Variance	0.72	0.57	0.15	0.05	0.06	0.34
Cronbach’s alpha	0.90	0.88	0.67	–	–	0.64

*Note.* WMC = working memory capacity; MW = mind wandering. *p < .05; ***p < .001

### Structural equation modeling

A structural equation model was subsequently estimated to test the hypotheses shown in Fig. 1. In this model, WMC, interest, topic familiarity, and comprehension were set up as latent variables. The latent variables referring to the former three constructs were represented at the level of individual items, whereas comprehension was indicated by three item parcels instead of treating each comprehension question as a single indicator (see Little, Cunningham, Shahar, & Widaman, 2002, for a detailed discussion about the benefits of parceling when there are many indicators for a construct). Voluntary and involuntary MW were modeled as manifest variables. Because interest and topic familiarity were measured on ordinal scales, the corresponding variables were treated as categorical predictors. Finally, to account for the multilevel structure of the data—texts were nested in participants—intercepts were allowed to vary per participant.

**Fig. 2:**
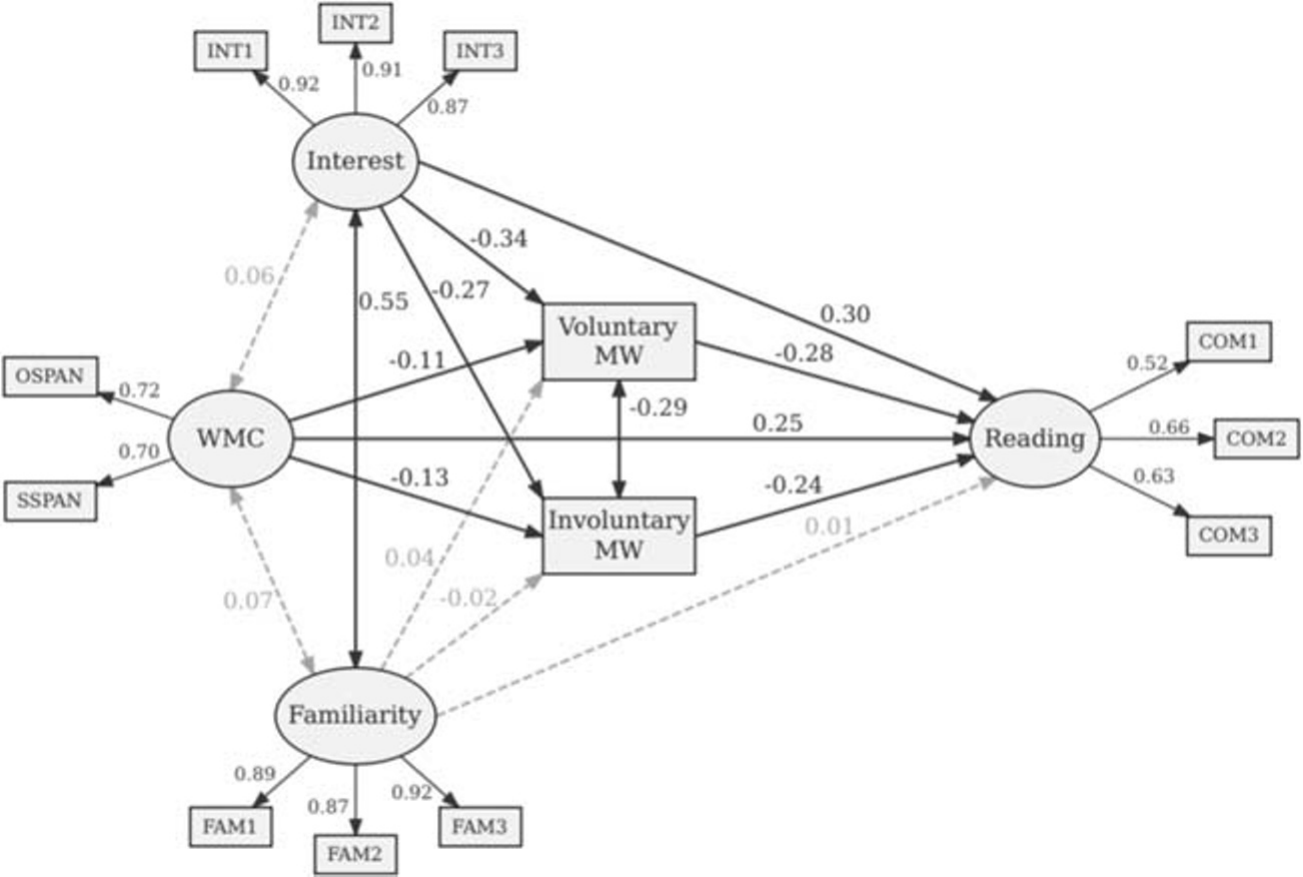
Structural equation model with estimated standardized coefficients. Grey dashed lines indicate nonsignificant paths

The fit of the model was good according to common criteria (χ^2^ = 129.718, *p* < .001, *df* = 52, Comparative Fit Index (CFI) = .990, Tucker-Lewis Index (TLI) = .986, Root Mean Error of Approximation (RMSEA) = .053), and the signs of the estimated path coefficients corresponded to our predictions (see Table 2 and Fig. 2). Most importantly, WMC was positively related to reading comprehension and negatively related to both forms of MW. Furthermore, the positive effect of WMC on comprehension was partially mediated by both forms of MW (see Table 3). These results support the main hypothesis that low-WMC individuals were more likely to engage in voluntary MW and that this partly explains their lower task performance. Furthermore, we replicated prior research showing that high-WMC individuals tend to suppress involuntary MW episodes more strongly than low-WMC individuals, and this explains another part of the variance in task performance. As regards the remaining results, interest was negatively related to both voluntary and involuntary MW, and, furthermore, the effect of interest on comprehension was mediated by both forms of MW (see Table 3). Somewhat surprisingly, topic familiarity did not significantly predict MW or comprehension in this model, despite the earlier reported significant correlations. Taking into account that interest and topic familiarity highly correlated (*r* = .55), both variables seemed to explain largely overlapping parts of the variance in MW and reading comprehension, although the two constructs were empirically separable.^5^

**Table 2:** Standardized coefficients, standard deviations, expected signs of the coefficients, and *p* values for each path of the estimated model

Directional path coefficients					
Dependent variable	Predictor variable	*b*	*SD*(*b*)	Expected	*p* value
Comprehension	Interest	0.30	0.07	*b* > 0	<.001
	Topic familiarity	0.01	0.06	*b* > 0	.834
	WMC	0.25	0.05	*b* > 0	<.001
	Voluntary MW	−0.28	0.06	*b* < 0	<.001
	Involuntary MW	−0.24	0.06	*b* < 0	<.001
Voluntary MW	Interest	−0.34	0.04	*b* < 0	<.001
	Topic familiarity	0.04	0.04	*b* < 0	.315
	WMC	−0.11	0.05	*b* < 0	.026
Involuntary MW	Interest	−0.27	0.05	*b* < 0	<.001
	Topic familiarity	−0.02	0.06	*b* < 0	.712
	WMC	−0.13	0.05	*b* < 0	.007
Bidirectional path coefficients					
Variable 1	Variable 2	*b*	SD(b)	Expected	*p* value
Interest	WMC	0.06	0.05		.229
Interest	Topic familiarity	0.55	0.03	*b* > 0	<.001
WMC	Topic familiarity	0.07	0.06		.203
Voluntary MW	Involuntary MW	−0.29	0.06		<.001

*Note.* WMC = working memory capacity; MW = mind wandering

**Table 3:** Standardized coefficients, standard deviations, expected signs of the coefficients, and *p* values for the mediator paths in the estimated model

Predictor variable	Mediator variable	*b*	*SD*(*b*)	Expected	*p* value
WMC	Voluntary MW	0.03	0.02	*b* > 0	.035
WMC	Involuntary MW	0.03	0.01	*b* > 0	.016
Interest	Voluntary MW	0.10	0.02	*b* > 0	<.001
Interest	Involuntary MW	0.06	0.02	*b* > 0	.001
Topic familiarity	Voluntary MW	−0.01	0.01	*b* > 0	.304
Topic familiarity	Involuntary MW	0.01	0.01	*b* > 0	.710

*Note.* WM = working memory; MW = mind wandering

**Table 4:** Bivariate correlations and descriptive statistics the manifest variables

Construct	Interest			Topic familiarity			WMC		Mind wandering		Comprehension		
Item	INT1	INT2	INT3	FAM1	FAM 2	FAM 3	OSPAN	SSPAN	VOL	INV	COM1	COM2	COM3
INT1	–												
INT2	0.76	–											
INT3	0.74	0.72	–										
FAM1	0.34	0.37	0.32	–									
FAM2	0.41	0.48	0.40	0.68	–								
FAM3	0.36	0.40	0.34	0.77	0.70	–							
OSPAN	0.11	0.08	0.046	0.01	0.11	0.03	–						
SSPAN	0.05	−0.03	−0.05	−0.00	0.07	0.06	0.50	–					
VOL	−0.30	−0.27	−0.26	−0.08	−0.17	−0.12	−0.06	−0.13	–				
INV	−0.29	−0.22	−0.24	−0.17	−0.15	−0.13	−0.14	−0.08	−0.15	–			
COM1	0.22	0.18	0.18	0.06	0.11	0.11	0.11	0.26	−0.24	−0.16	–		
COM2	0.29	0.28	0.26	0.13	0.21	0.17	0.14	0.14	−0.21	−0.21	0.41	–	
COM3	0.29	0.25	0.27	0.10	0.20	0.15	0.15	0.15	−0.24	−0.22	0.34	0.36	–
Mean	1.70	1.55	1.43	1.28	1.53	1.30	0.70	0.57	0.13	0.27	1.82	1.61	1.86
Variance	0.86	0.86	0.82	0.93	0.88	0.81	0.04	0.03	0.05	0.06	0.82	0.89	0.92
Skew	−0.23	−0.05	−0.07	0.20	−0.14	0.14	−1.17	−0.28	2.12	0.81	−0.36	−0.08	−0.38
Kurtosis	−0.80	−0.85	−0.82	−0.91	−0.87	−0.79	1.09	0.06	4.43	0.41	−0.67	−0.90	−0.86
Range	0–3	0–3	0–3	0–3	0–3	0–3	0–1	0–1	0–1	0–1	0–3	0–3	0–3
Minimum	0	0	0	0	0	0	0.03	0.07	0	0	0	0	1
Maximum	3	3	3	3	3	3	1	1	1	1	3	3	3

INT = interest; FAM = topic familiarity; OSPAN = operation span; SSPAN = symmetry span; VOL = voluntary mind wandering; INV = involuntary mind wandering; COM = comprehension. Note that the items COM1-3 represent parcels that were created by grouping together each three items from the multiple-choice test

## Discussion

The current study investigated the relations between WMC, voluntary/involuntary MW, and performance in a reading comprehension task. The most important and novel results in comparison to prior research (Ju & Lien, 2018; Robison & Unsworth, 2018; Soemer & Schiefele, 2019) are (1) that high-WMC individuals reported fewer voluntary MW episodes compared with low-WMC individuals, (2) that the negative relation between WMC and voluntary MW remained after controlling for interest, and (3) that voluntary MW partially mediated the effect of WMC on reading performance. Moreover, we also replicating previous findings showing that involuntary MW impairs reading comprehension and partially mediates the positive effect of WMC on comprehension (e.g., McVay & Kane, 2012; Soemer & Schiefele, 2019).

Our preferred interpretation of the inverse association between voluntary WM and WMC is that low-WMC individuals experience greater difficulties while extracting facts from a text and integrating them into a coherent mental model (Swanson et al., 2009), and that these difficulties may especially encourage low-WMC individuals to skip parts of the text, or give up completely, and deliberately pursue less stressful thoughts instead. Related to this explanation, prior research has demonstrated an inverse association between readability, a measure of objective text difficulty, and voluntary MW (Soemer & Schiefele, 2019), suggesting that the more difficult a text, the more likely it becomes that an individual engages in voluntary MW. Here, although our texts were of overall moderate readability, they may have nevertheless been relatively difficult for low-WMC individuals. As a consequence, these individuals may have been more inclined to deliberately start a MW episode or deliberately continue a MW episode that started as an involuntary episode.

An alternative explanation is related to self-efficacy beliefs—that is, an individual’s expectation of being successful in a given task. Prior research suggests that low-WMC individuals have lower self-efficacy beliefs (Linnenbrink & Pintrich, 2003), and it may therefore be argued that they are less willing to engage and persist in a task or refocus their attention on the text when they become aware of their (involuntary) MW. Finally, prior research suggests that low-WMC individuals are less accurate with regard to meta-cognitive judgments of their performance in demanding tasks, because they lack the capacity to perform the task and carry out the judgments at the same time (Komori, 2016). We suggest that low-WMC individuals may also more easily misjudge the impact of voluntary MW on reading comprehension, compared with high-WMC individuals, and therefore allow more voluntary MW to take place during task execution.

Whereas our results deviate from prior research insofar as we obtained, for the first time, significant effects of WMC on voluntary MW, they do not generally contradict the control failures account of MW (Kane & McVay, 2012). To reiterate, this account states that WMC reflects the ability to control the focus of attention and inhibit external and internal distraction. Accordingly, because high-WMC individuals are better at suppressing internal distraction, they report less (involuntary) MW, and this partially explains their better task performance. That being said, the negative relation between WMC and voluntary MW as well as the negative effect of voluntary MW on comprehension were not explicitly predicted by the control failures account, as this account is mainly concerned with involuntarily MW (Kane & McVay, 2012, pp. 348–349). In line with more recent proposals regarding the distinction between voluntary and involuntary MW (Seli et al., 2015; Seli et al., 2016), we argue that high-WMC individuals use their better executive control capabilities not only for suppressing involuntary MW episodes but also for limiting their level of voluntary MW whenever this is required for successfully carrying out a given task (Rummel & Boywitt, 2014).

Finally, it is also particularly noteworthy that voluntary MW impaired comprehension even though our model controlled for interest, an important motivational variable. Thus, in contrast to prior research arguing that voluntary MW might be more closely related to motivational factors rather than to executive control (Soemer & Schiefele, 2019), interest and voluntary MW seemed to explain partially separate parts of variance in reading comprehension. In sum, the present results support the idea that executive control (i.e., WMC) is used to regulate thought content both by suppressing involuntary occurring MW and by limiting voluntary MW.

### Final note

It may be argued that such conclusions should be drawn with great caution given the number of nonsignificant results in prior studies. Although we have argued earlier that these nonsignificant results do not tell us much about the sign and the strength of the relation between WMC and voluntary MW, it seems nevertheless meaningful to incorporate estimates from prior research into our analysis to address concerns of replicability. We therefore carried out a Bayesian reanalysis of our structural equation model (SEM) using parameter estimates from a similar previous study (Soemer & Schiefele, 2019) as priors for our model parameters, and provide the results of this reanalysis as a supplement. To foreshadow the results, we found the Bayesian reanalysis to be broadly consistent with the traditional analysis reported above. In particular, the negative relations between WMC and the two forms of MW shown in the traditional analysis remain statistically reliable in the Bayesian reanalysis.

### Electronic supplementary material

ESM 1 (DOCX 33 kb)

## Appendix: Mind-wandering instructions (approximate translation)

(Slide 1) “Do you recognize this situation? You are sitting in front of your desk reading a text. Your eyes move from left to right, scanning every word, sentence, and paragraph. After some time, however, you start thinking about something that has nothing to do with the text. Perhaps, you don’t notice it immediately; perhaps your eyes are still moving naturally across the text as if you were still focusing on reading. But at some point, you become aware that your thoughts have been drifting off and that you have to reread the previous paragraph.”

(Slide 2) “Every human being experiences such task-unrelated thoughts under certain circumstances while carrying out certain tasks. This is a natural phenomenon called ‘mind wandering’ in psychological research. In this study, we are interested in the role of intention in mind wandering.”

(Slide 3) “Sometimes, we unintentionally drift off, despite our best effort to focus on a task such as reading a text. We call this ‘involuntary mind wandering,’ because it happens although we actually don’t intend to mind wander. Sometimes we drift off deliberately because we don’t want to focus on the task in that specific moment. We call this ‘voluntary mind wandering.’”

(Slide 4) “In this study, we will ask you to read a number of texts dealing with different subject areas. From time to time during reading, we will interrupt you and ask you to indicate whether you were focusing on the text just prior to the interruption or, instead, voluntarily or involuntarily engaging in mind wandering.”

(Slide 5) “When we interrupt you, the following question will appear on the screen:

*What were you just thinking about? “(1) About something related to the text”; “(2) About something unrelated to the text (voluntarily)”: “(3) About something unrelated to the text (involuntarily).”*

Please answer this question with the keys 1–3. Of course, sometimes there is no clear relation between “related” and “unrelated” thoughts, but the general rule is as follows: If the thoughts you experienced aimed at promoting comprehension, please choose (1). Thoughts that did not aim at promoting text comprehension count as “unrelated to the text” (2, 3).

(Slide 6). “Please be totally honest with regard to the mind wandering reports. There really is no ‘right’ or ‘wrong’ answer.”

## Footnotes

1 Of course, the internal stream of thought might itself be influenced by external events.

2 Alternative labels are “intentional” or “deliberate” for voluntary MW, and “spontaneous” or “unintentional” for involuntary MW. For convenience, here we only use the terms “voluntary” and “involuntary” MW.

3 For involuntary forms of MW, there is evidence suggesting that a negative relation with WMC only shows up if a task is sufficiently demanding (e.g., Rummel & Boywitt, 2014). This could likewise be the case with voluntary MW.

4 There were 10 instances of no-shows or equipment errors. Any partial data was discarded completely without analysis and a replacement was scheduled to reach the target sample size of 180 in these cases.

5 The fit of a two-factor model with separate factors for ‘interest’ and ‘familiarity’ was substantially better (χ^2^ = 52.718, *p* < .001, *df* = 8, CFI = .995, TLI = .990, RMSEA = .102) than the fit of a one-factor model (χ^2^ = 577.013, *p* < .001, *df* = 9, CFI =.933, TLI = .888, RMSEA = .343) or a two-factor model with the correlation between the two factors set to 1.0 (χ^2^ = 801.362, *p* < .001, *df* = 9, CFI = .910, TLI = .849, RMSEA = .405.
